# FABP9 Mutations Are Not Detected in Cases of Infertility due to Sperm Morphological Defects in Iranian Men

**Published:** 2013-12-22

**Authors:** Javad Jamshidi, Farkhondeh Pouresmaeili, Hossein Darvish, Mir Davood Omrani, Eznollah Azargashb, Mohammad Reza Sadeghi, Niknam Lakpour

**Affiliations:** 1 Department of Biochemistry, Fasa University of Medical Sciences, Fasa, Iran; 2 Department of Medical Genetics, Infertility and Reproductive Health Research Center (IRHRC), Faculty of Medicine, Shahid Beheshti University of Medical Sciences, Tehran, Iran; 3 Department of Community Medicine, Faculty of Medicine, Shahid Beheshti University of Medical Sciences, Tehran, Iran; 4 Monoclonal Antibody Research Center, Avicenna Research Institute, ACECR, Tehran, Iran; 5 Nanobiotechnology Research Center, Avicenna Research Institute, ACECR, Tehran, Iran

**Keywords:** *FABP9*, Mutation, Fertility, Sperm

## Abstract

**Background:**

Fatty acid binding proteins (FABPs) are members of the intracellular li- pid binding protein (iLBPs) family and most of them show tissue specific expression. *FABP9/PERF15* (Perforatorial15) is the male germ cell-specific fatty acid-binding pro- tein. It was first identified as the major constituent of the murine sperm perforatorium and perinuclear theca. To date, investigations in mice have demonstrated that this protein has a role in the male reproductive system, especially in spermatogenesis. Also, it has been reported that *FABP9* can protect sperm fatty acids from oxidative damage. Recently it was shown that it can affect sperm morphology in mice. Based on these findings, we designed a study to evaluate if mutations of this gene can affect sperm morphology in humans.

**Materials and Methods:**

In this case-control study, DNA was extracted from peripheral blood of 100 infertile males with normal sperm count but with a number of morphologi- cally abnormal sperms in their semen that was above normal. Four exons and one intron of the *FABP9* gene were amplified by polymerase chain reaction (PCR), re-sequenced and then analyzed for mutation detection.

**Results:**

We did not detect any mutation in any area of the four exons, intron 3 and splice sites of *FABP9* gene in any of the studied 100 samples.

**Conclusion:**

There was no mutation in the exonic regions and the poor sperm mor- phology. However, we didn’t analyze the promoter, intron 1 and 2 to establish conclusions regarding the association of these genic regions and sperm dysmor- phology.

## Introduction

Fatty acid binding proteins (<italic>FABPs</italic>) are a group of proteins which can bind to fatty acids, specially long chain (C12-20), which differ in their selectivity, affinity and binding mechanisms (1, 2). They are members of the intracellular lipid binding proteins (iLBPs) family and most of them show tissue specific expression. *FABPs* are structurally cytosolic proteins and demonstrate strong evolutionary conservation (3). To date, 10 FABP coding genes have been identified in the human genome. Numerous functions have been demonstrated for *FABPs* such as intracellular fatty acid trafficking, fatty acid metabolism, signal transduction, cell growth and differentiation, and regulation of gene expression (1, 3-6). *FABP9* (also known as <italic>T-FABP</italic> or <italic>PERF15</italic>) is a poorly understood member of the FABP family which is located on chr. 8q21.13. *FABP9* was first identified as a major component of the rat sperm perinuclear theca (7). *FABP9* has the highest homology with myelin P2, and it has been suggested that it may share some functions with the myelin P2 protein. *FABP9* is expressed during spermatogenesis in mammalian testis (7, 8) and it has been proposed that *FABP9* has a role in the apoptosis of these cells (9). Another study has demonstrated that it can protect sperm fatty acids from oxidation, thus maintaining their ability to fertilize oocytes (10).

The mammalian sperm is a vital cell in reproduction and its intact function is essential. There are many problems that can affect sperm physiology; it can be functional defects, numerical defects or morphological defects of this cell. A recent study by Selvaraj et al. (11) demonstrated that mice deficient in *FABP9*, are fertile but they show increased morphological defects in their sperm structure relative to wild type mice. There have been no investigations regarding the role and function of *FABP9* in humans however, according to the evidence in mice; *FABP9* is likely to have a role in the male reproductive system, especially in the function and the structure of sperm. In the present study, we hypothesized that this protein has a role in human spermatogenesis and mutations in this gene may cause sperm morphological defects. Our study was designed to evaluate this hypothesis.

## Materials and Methods

### Study population

In this case-control study, normal and patient human samples were collected from the individuals according to a formal agreement when referring to Avicenna Research Institute. All human studies have been reviewed by the appropriate Ethics Committee.

From patients who were referred to the Avicenna fertility and infertility center, Tehran, Iran, 100 men who had fulfilled our criteria were randomly selected. These men were infertile and their sperm count was normal but their semen analysis showed more than 30% (according to the WHO criteria) morphologically defective sperm. These men had normal karyotype and the cause of infertility and defective sperm morphology was unknown.

Men with anatomical defects in the reproductive system, genetic syndromes (e.g. XXY), oligozospermia and azospermia, as well as couples for which the cause of infertility was a female factor were excluded from our study. Anthropometric parameters including age, body height and weight were all recorded. A complete semen analysis was carried out for each subject.

A population of 100 fertile men who had children was also selected as the control group. Since first we tested infertile cases and found no mutation in the gene, there was no need to study the same regions of the gene in fertile healthy subjects.

Sampling method was purposive, data was processed by statistical product and service solutions (SPSS16) and analyzed by χ^2^ test.

### DNA extraction

Peripheral blood samples was obtained from each subject. DNA was extracted from all samples using a salting out method.

### Polymerase chain reaction (PCR)

*FABP9* contains 4 exons and 3 introns. Three pairs of primers were designed to amplify all 4 exons. As exons 3 and 4 and the intron between them are short in length, only one pair of primers was designed to amplify this sequence. Primers were designed using Primer blast software (http:// www.ncbi.nlm.nih.gov/tools/primer-blast). The primers and the size of the amplified sequence are as follows: for exon 1 forward: 5ʹ CTACTGGCAGCACCGTAATG 3ʹ, reverse: 5ʹ CCCTAGGATCACAAAAGGAAG 3ʹ, 259 bp, for exon 2 forward: 5ʹ GAAAATTGACTTCCAGAGTGATTG 3ʹ, reverse: 5' AACCATGCCTAACCACCTT 3', 306 bp, for exon 3 and 4: forward: 5' TGTGGTCTCATGTAGGTTAGAAGG 3', reverse: 5' TCTTCAGGTACCAGTTCCTTGTC 3', 440 bp. The PCR reaction was performed in 25 μl volume containing 100-300 ng of extracted DNA, 1X PCR buffer (50 mM KCl; 10 mM Tris-HCl; 1.5 mM MgCl<sub>2</sub>), 2 mM MgCl_2_, 200 μM dNTP mix and double distilled water was added up to 25 μl with 1 U of Taq DNA polymerase (super Taq DNA polymerase, Gen Fanavaran co., Tehran, Iran) and 0.4 μM of each oligoneucleotide primer. PCR products were loaded on 1% agarose gel followed by ethidium bromide staining to confirm the detected amplified fragments.

### Sequencing

After confirming the PCR reaction and purification, PCR products were sequenced using an ABI 3730XL DNA Analyzer manufactured by Applied Biosystems (Bioneer, South Korea). The results of DNA sequencing were analyzed by Chromas software (version 2.33 Technelysium Pty Ltd) and the results were compared with the reference sequence using national center for biotechnology information (NCBI) blast software (http://blast.ncbi.nlm. nih.gov).

### Polymerase chain reaction-restriction fragment length polymorphism

We also analyzed the frequency of a single nucleotide polymorphisms (SNP, rs28485205) located at intron 3 of the *FABP9* gene between case and control groups using the polymerase chain reaction- restriction fragment length polymorphism (PCR-RFLP) method. The primers were the same as primers used for amplification of exons three and four. 5 μl of each PCR product was digested with 1U of AluI restriction enzyme (Jena Bioscience EN-101S) at 37˚C for about 1 hour. The enzyme cuts when the nucleotide in this site is G but not when there is an A. The original PCR product is 440 bp lengths, therefore, if the enzyme cuts, there will be 2 fragments of 306 bp and 134 bp.

## Results

**Fig 1:**
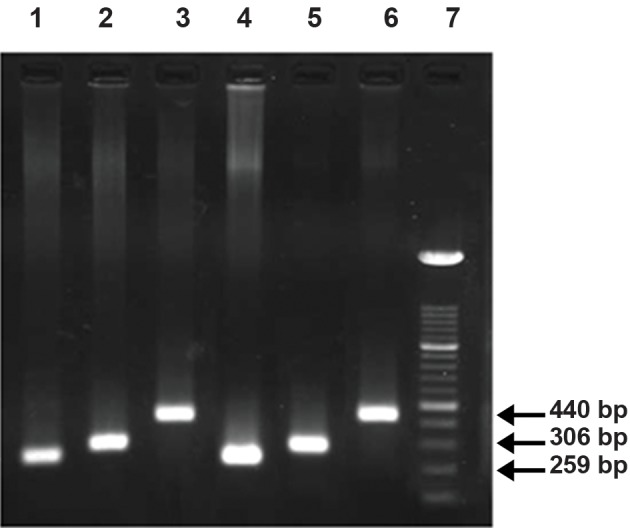
PCR amplification. Fragments of the gene were detected after electrophoresis on 1% agarose gel. Columns 1and 4: exon1, columns 2 and 5: exon2, and columns 3 and 6: exons3, 4 and intron3. Column 7 is 100 bp size marker.

**Fig 2:**
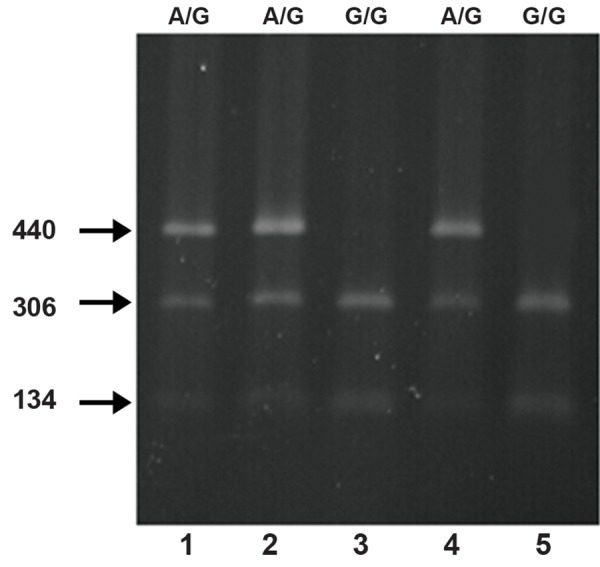
PCR-RFLP result. PCR products were digested by AluI and electrophoresed on 2% agarose gel stained by ethidium bromide. Lanes 1, 2 and 4 are heterozygotes for the polymorphism A>G with three distinct bands of 440, 306 and 134 bp. Lanes 3 and 5 are homozygote pattern for the single nucleotide polymorphism with two different bands of 306 and 134 bp.

The result of PCR amplification of the whole gene except introns 1 and 2 and the promoter region was three distinct fragments (Fig 1). A 259 bp segment contains exon1; the 306 bp fragment bearing exon2 and the 440 bp contains the exons 3&4 in addition to the third intron sequences of *FABP9*. PCR-RFLP for SNP (rs28485205) in the third intron showed that the region was digested by AluI restriction enzyme resulted in three different bands in heterozygote pattern but produced only two bands of distinct molecular weight in homozygote samples (Fig 2).

All 4 exons of *FABP9* gene were sequenced in each of 100 patient samples. No mutation was detected in the four exons, intron 3 and splice sites of the *FABP9* gene in these 100 samples. We detected three SNPs in the sequenced regions of the gene in our samples in comparison to the reference gene sequence. The frequency and details of these SNPs are shown in table 1.

### Variants study

The present Ensemble (http:/www.ensemble. org) database informing of 31 different variants in the sequenced regions of the gene with 5, 10 and 16 variants with the first, second and third primer pair regions respectively. Three of the variants were SNPs and were seen in our samples as rs62513549 in intron 1, rs28485205 and rs75129444 in intron3. The allele frequency was calculated for and compared between the three single nucleotide polymorphisms as is demonstrated in table1.

The SNP rs28485205 allele frequency in infertile men was compared with a normal (fertile) group of 100 fertile individual by χ^2^ test with no significant difference (p=0.458). Also, the SNP genotype frequency was not significantly different between the two groups (p=0.257). The results are shown in table 2.

**Table 1:** Genotypes and allele frequency distribution in the detected SNPs in our samples


SNP	Genotype %			Allele frequency	

**rs62513549**	C/C	/G	G/G	C	G
	88 %	11 %	1 %	93.5 %	6.5 %
**rs28485205**	G/G	G/A	A/A	G	A
	80 %	18 %	2 %	89 %	11 %
**rs75129444**	G/G	G/A	A/A	G	A
	99 %	1 %	0 %	99.5 %	0.5 %


**Table 2:** Comparison of genotypes and allele frequencies of rs28485205 in fertile and infertile groups


Group	Genotype %			Allele frequency	
	G/G	G/A	A/A	G	A

**Fertile**	86 %	14 %	0 %	93 %	7 %
**Infertile**	80 %	18 %	2 %	89 %	11 %


## Discussion

Previous studies have shown that *FABP9* has a possible role in sperm morphology and function (12, 13). This protein is one of the members of *FABP* gene family which is only present in the genome of mammals (7, 14). Moreover, it seems that the protein could have an influence on programmed death of spermatocytes and their development as well as a protection role to oxidation towards sperm fatty acids which in turn increases fertility potential (10). A very recent study, determined that the absence of the protein affects mice sperm morphology (11). Based on evidence that emphasizes that *FABP9* could be effective in spermatogenesis, sperm shaping and perhaps in human fertility, in this study, we investigated the presence of potential mutations in *FABP9* of infertile men which could affect the morphology and fertility/ viability of human sperm. Although, we did not observe any mutation in the coding regions of study and have not investigated other relevant regions of the gene, promoter, intron 1 and 2, yet, the questions remain as to the role of the *FABP9* product in human reproduction and its location in the human testis.

However, there are genes which are identified to be involved in the sperm morphology development (globozoospermia, 15) and it seems they could be used as gene markers. Therefore, it appears that further genetic investigation of infertility and finding candidate genes for genetic markers for diagnosis of male infertility may be necessary.

Testis is a composed gonad of different compartment and cell types. Although it is shown that *FABP9* is expressed in testis, there is no evidence to show the specific location of the gene expression in human testis. One possibility could be that the protein is expressed in a different cell type of human testis like interstitial tissue cells, compare to the mice *FABP9* expression in spermatozoa. Then, regarding the importance of interstitial tissue protein induction role on leydig cells which in turn are supportive for germ cells meiosis division, growth and synchronization, an indispensable unknown role could be contributed to *FABP9* in human spermatogenesis procedure, respectively. Our future plan for the human *FABP9* localization will clarify the fact.

While looking for mutations, we found several SNP in the patient population. The allele frequency of rs62513549 in Iranian population was close to Utah residents with Northern and Western European ancestry (CEU) and [Han Chinese in Beijing, China (CHB) + (Japanese in Tokyo, Japan) JPT] populations, rs 28485205 had a close similarity in allele frequency between our population and CEU while CHB + JPT and Yoruba in Ibadan, Nigeria (YRI) were different of either each other or of our population or of CEU. The third SNP was rs75129444 which showed to be close in allele frequency between Iranian and CEU populations.

Presently, due to restricted investigation on human *FABP9* we are unable to establish the exact function of the protein and the related mechanism in humans. *FABP12*, one of the newly identified *FABPs* with expression in two different tissues, testis and retina. It shows most identity to *FABP9* in phylogeny, structure and expression pattern in testis in continuous sperm developmental stages (13). Therefore, one can further postulate that *FABP9*, like its counterpart, may have human tissue expression, presumably in different stage and/or different cell type of testis. Examining regions of the geneintrons 1 and 2 would offer a complete genetic analysis of this gene. The promoter region would be associated with gene-expression levels but, the complete promoter region for this gene is not well characterized. Further research will reveal any involvement of the gene in human fertility.

## Conclusion

This study showed no association between the examined *FABP9* exonic regions and sperm dysmorphology.
